# The significance of humorous tattoos

**DOI:** 10.1007/s12024-025-00941-9

**Published:** 2025-01-16

**Authors:** Roger W. Byard

**Affiliations:** 1 Adelaide School of Biomedicine, The University of Adelaide and Forensic Science SA, Adelaide, South Australia Australia; 2 Adelaide School of Medicine, Helen Mayo North, The University of Adelaide, Level 2, Room N237, Frome Road, Adelaide, SA 5005 Australia

**Keywords:** Tattoos, Humour, Joke, Contemporary trends

## Abstract

Tattooing refers to the process of creating indelible designs and texts in the human skin by introducing a variety of dyes. It has found for millennia in a range of societies. The purpose of tattooing has ranged from marking individuals of significant social standing such as chieftains in Polynesia, to those who are regarded as outcasts such as prostitutes and criminals in Europe. In recent years tattooing has gained considerable popularity in the West including tattoos that have merely had a humorous content, either as an image or text. These types of tattoos often utilize natural body features such as the umbilicus, natal cleft, genitalia, breasts or scars, and may contain simple but explicit messages or suggest possible underlying mental disturbance. While the significance of these types of tattoos and the profile of individuals who have them is at present unknown further study could identify sociological and forensic features specific to this subgroup.

## Introduction

Tattoos are very common in contemporary Western communities depicting a wide range of images and messages. The history of tattooing, however, goes back millennia to Neolithic times. An example is ‘Ötzi’ a hunter who lived around 3370 to 3100 BC whose body was found preserved in the glacial ice in the Tyrolean Alps still showing distinct tattooing [1]. Other tattooed mummified corpses have been found in Siberia, China and the Andes [2].

## Significance

Traditional tattoos have been applied for significant cultural and religious reasons, often marking individuals of ‘high degree’ such as warriors or chieftains, or being used for magical/therapeutic purposes [3]. This is exemplified amongst Polynesian groups of the South Pacific with waist to knee tattoos or *Pe’a* in Samoa, and facial tattoos or *Moko* amongst the Maori [2]. The reasons for tattooing change over time and in different cultures with skin markings being once found mainly in outcast groups in Europe such as criminals, prostitutes, gang members and prisoners [4]. The art of prison tattooing peaked in Russia with the skin of inmates sometimes being used as a canvas to record their entire criminal/incarceration history [5]. Modern day gang members still use certain symbols to demonstrate their allegiance to a particular group ranging from full body tattoos of the *Yakuza* in Japan to a cross between the first finger and thumb of the Pachuco street gang in California, United States [6, 7].

Tattoos depicting religious symbols such as a cross may signify a particular belief system and flags and images such as swastikas may be used to support a political ideology [8]. Tattoos may also be used to recognise family members or friends or to commemorate events such as marriages, births and deaths [9].

Alternatively, tattoos have been imposed on certain groups to mark social outcasts such as military deserters in eighteenth century Britain, replacing branding, and convicts sentenced to forced labour in nineteenth century France. More recently concentration camp inmates in Nazi Germany in World War II were tattooed with an identification numbers echoing a previous practice in Ancient Rome of using tattoos to identify criminals and slaves [7].

## Modern developments

In recent years tattooing has become extremely popular in the West with as many as one in five young adults and adolescents now having tattoos [10]. The sophistication of tattoos has also increased with fine line and quite extraordinary three dimensional designs by tattoo artists such as Jess Rix [11] contrasting with previous monochromatic primitive images [12]. Decorative tattoos have now become an art form.

While tattooing is not now associated with any particular cause or manner of death [4] analyses of specific subgroups with tattoos containing, for example, antisocial messages have shown an increased incidence of violent and unnatural deaths [8, 13, 14] However, given the ubiquity of tattoos in the general population any trends or features of particular smaller subgroups will in all likelihood be lost. Thus, review of individuals with tattoos that contain either a humorous image or text may identify unique features.

## Humorous tattoos

Tattoos with a purely humorous focus that may be encountered in forensic cases vary greatly in style and may be polychromatic and well-executed or monochromatic and unsophisticated. Not uncommon ones have included ‘I’m tired’ in letters across the backs of the toes or a previous partner’s name on the buttocks. Sexual themes are not unusual including arrows pointing to the genitalia with sometimes explicit instructions/messages. Internet websites have further examples.

**Fig. 1:**
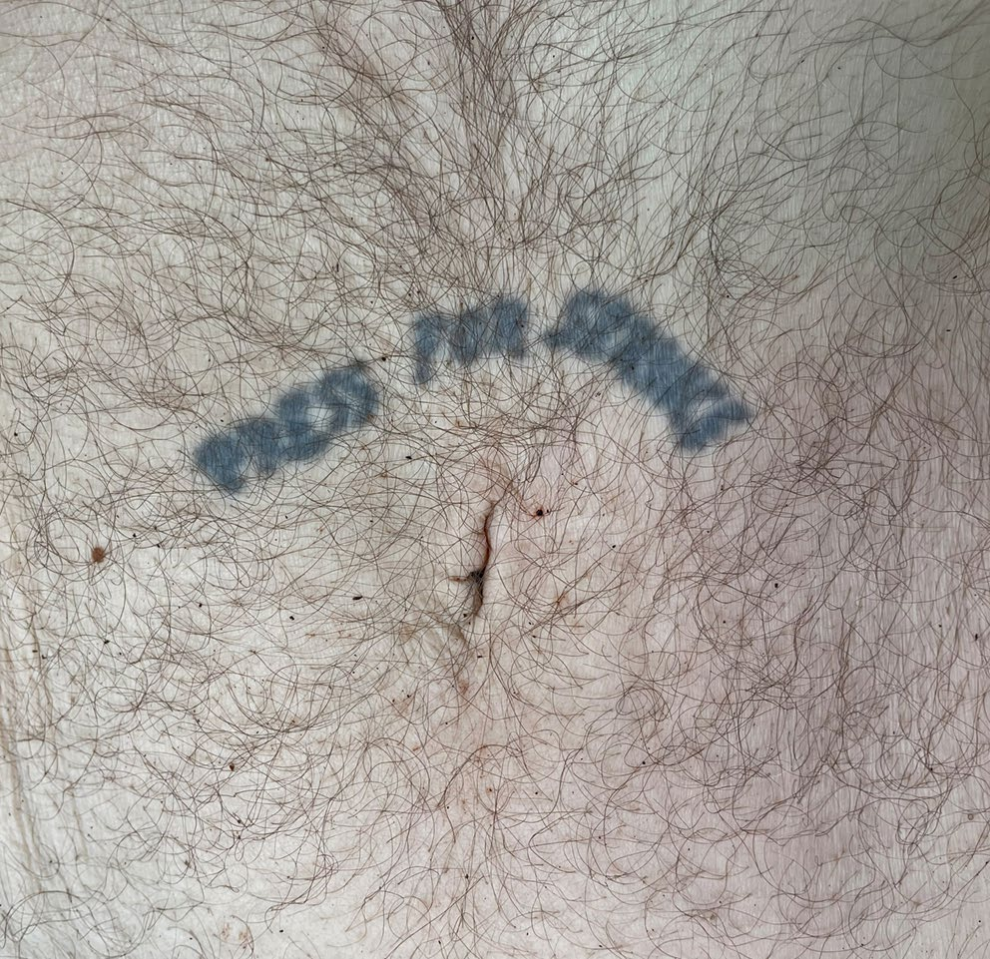
A primitive monochromatic tattoo with ‘Press for Service‘ above the umbilicus

**Fig. 2:**
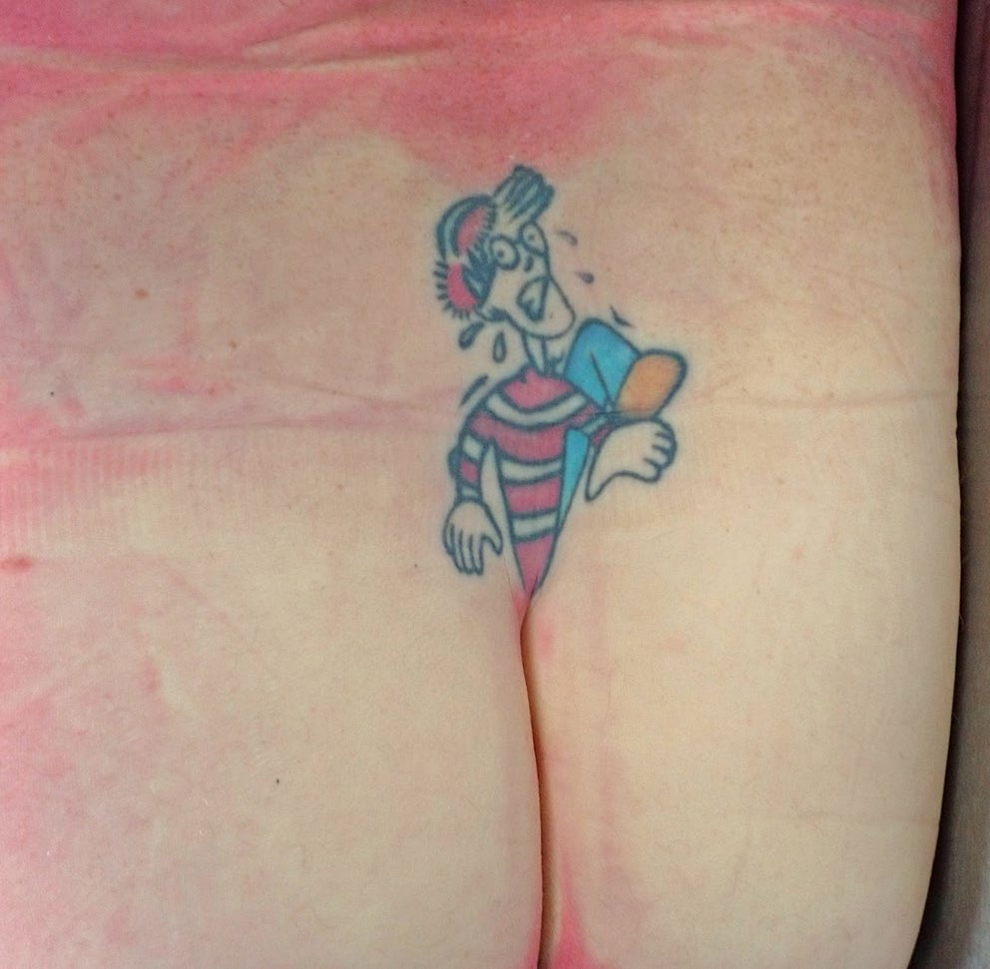
A cartoon character appearing to be trapped in the upper part of the natal cleft

**Fig. 3:**
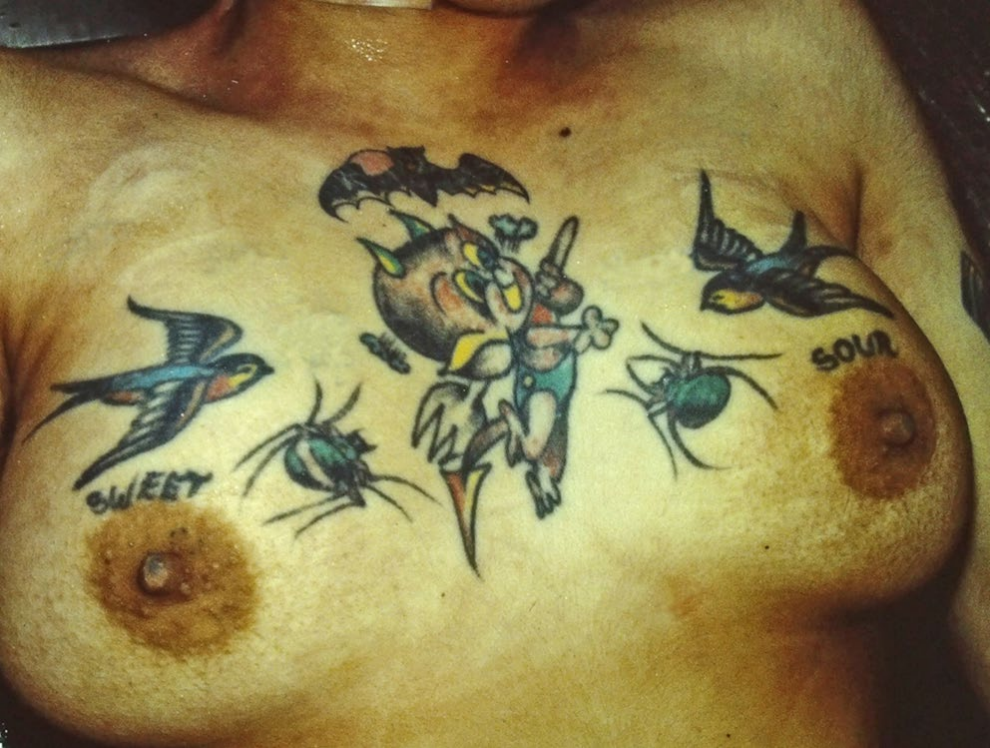
A cartoon character with ‘sweet’ and ‘sour’ on alternate breasts

**Fig. 4:**
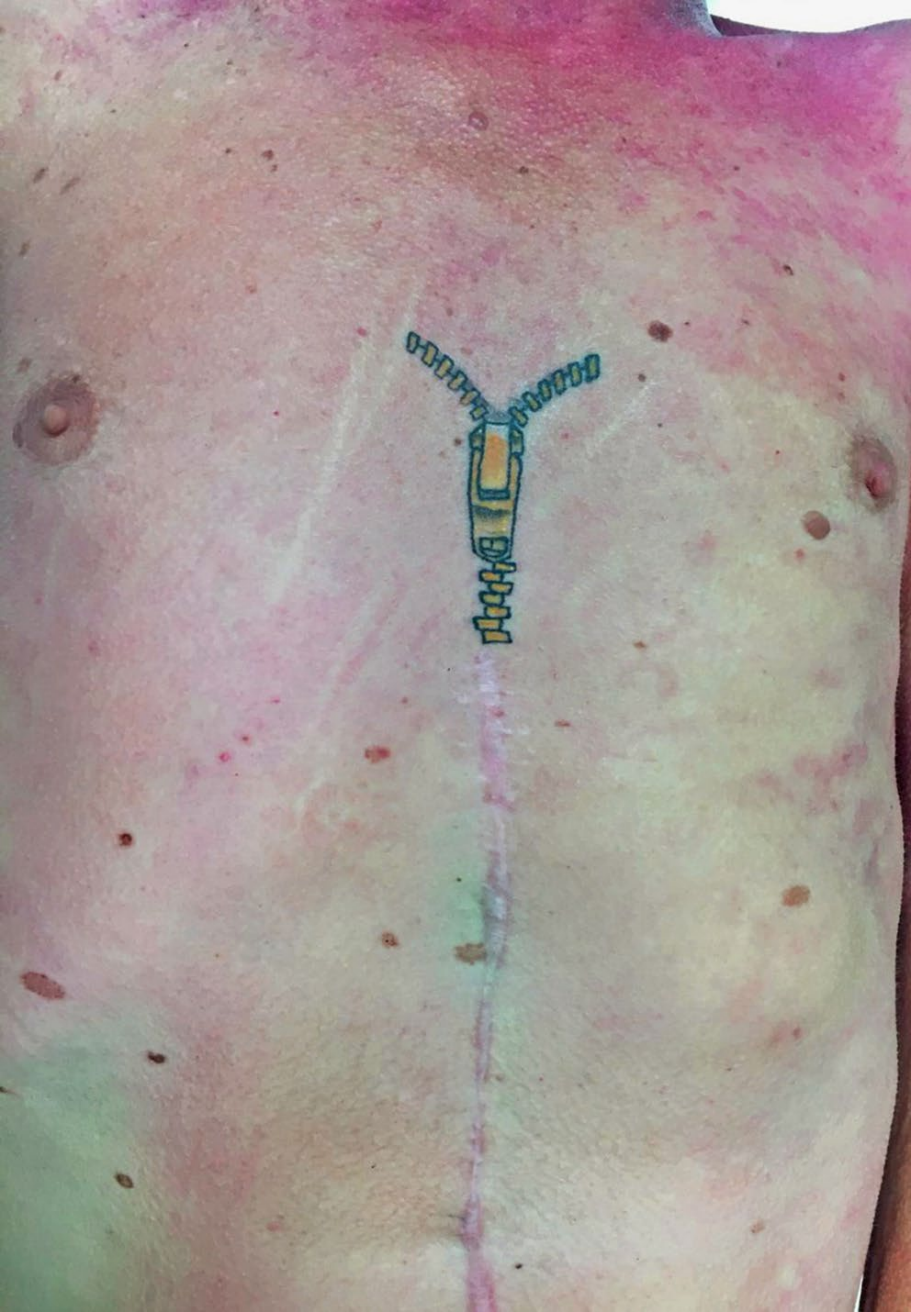
A well-executed polychromatic image of a zipper added to the upper part of a healed sternotomy scar

**Fig. 5:**
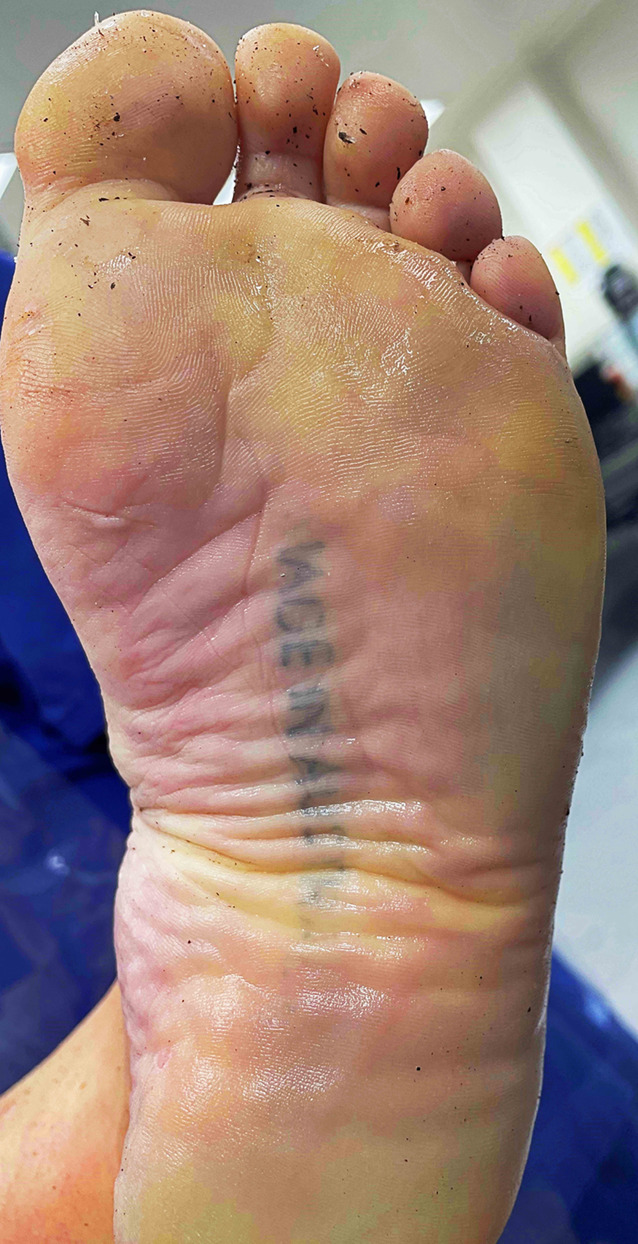
A fading indistinct tattoo with ‘Made in Australia’ written on the sole of a foot was a humorous way to record the country of origin of the decedent

**Fig. 6:**
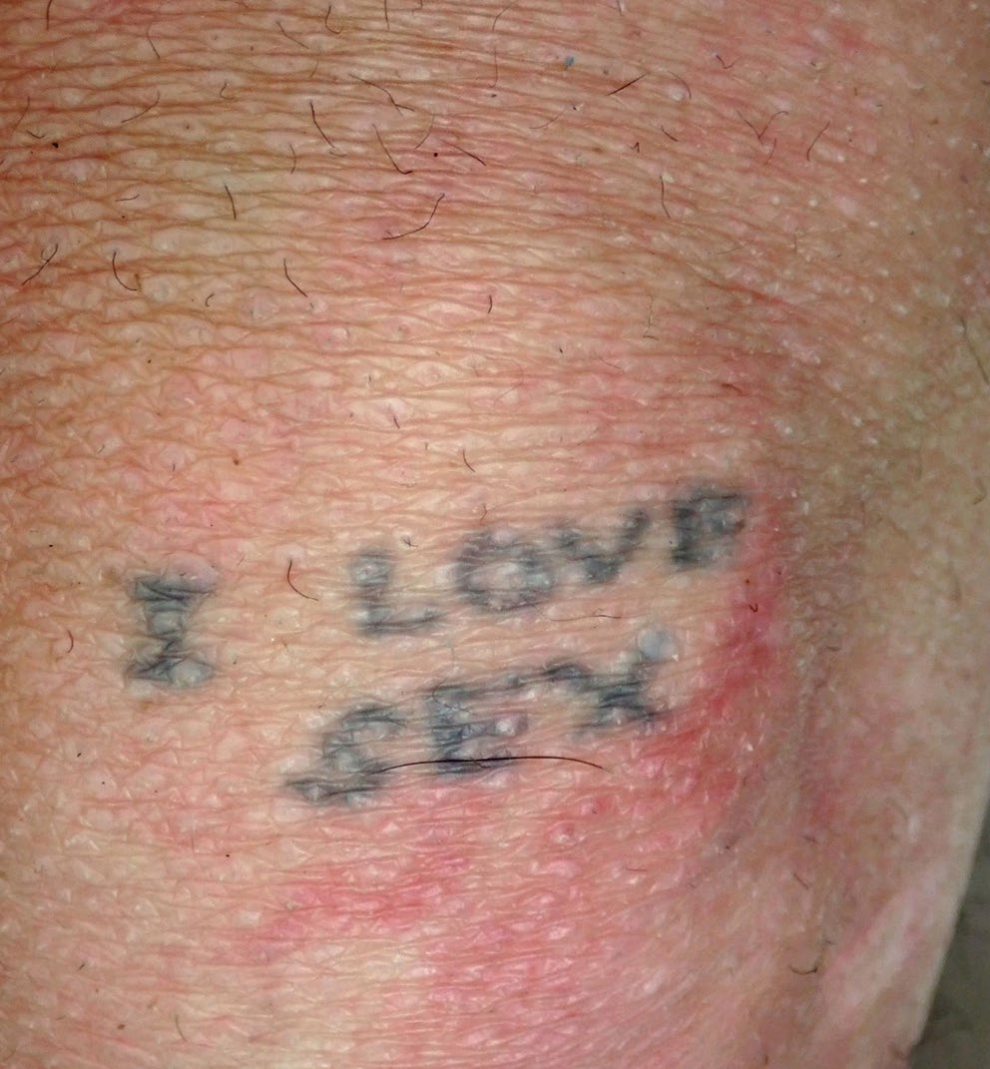
A primitive tattoo on the arm stating ‘I love sex’

**Fig. 7:**
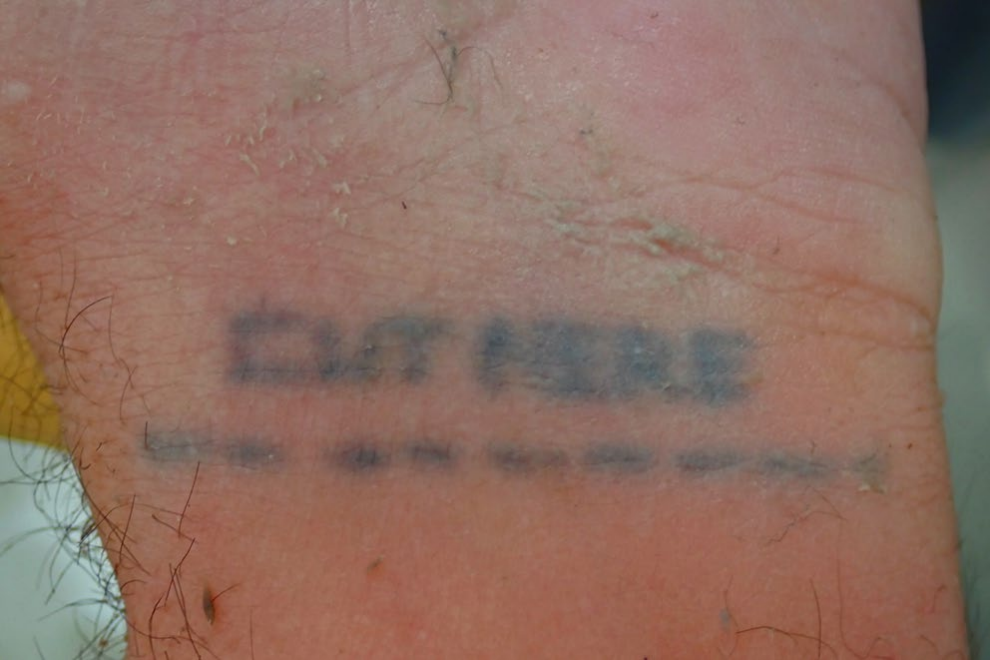
This instruction for suicidal wrist cutting with ‘Cut Here’ may give an indication of underlying psychological illness through dark humour

A selection of images taken from the Pathology Archive at Forensic Science South Australia demonstrate the range and content of images that may be found. These tattoos often utilize natural body features such as the umbilicus (Fig. 1), natal cleft (Fig. 2), genitalia, breasts (Fig. 3) or scars (Fig. 4). They may be in locations that can only be seen if someone is lying down (Fig. 5) or may contain simple but explicit messages (Fig. 6), or be tinged with dark humor suggesting possible underlying mental disturbance (Fig. 7). All but one of the depicted tattoos were from male decedents.

The reason for tattoos with a humorous theme or message in recent years is unclear but may be a reflection of a general relaxation in what may have been previously perceived as unacceptable or offensive, or of a greater willingness to expose such designs/texts. Although it would be informative to have psychological profiles of those who opt for such decoration, as permanently marking the body with comical images seems to form a specific subset of contemporary Western tattooing, there has been minimal study of this phenomenon.

As analyses of subgroups of individuals having specific types of tattoos has shown that there may be differences in the cause and manner of death, further study of cases with humorous tattoos may be informative. Unfortunately the numbers of cases in the present study (*N* = 7) are too small to draw any meaningful conclusions, however, larger population-based studies may identify sociological or forensic features of interest. Pertinent features to be evaluated in larger studies would be the age and sex of the decedents, drug usage or gang membership, the cause and manner of deaths, and the association with any other types of tattoos.

## Data Availability

None.
